# Virtual reality for assessing stereopsis performance and eye characteristics in Post-COVID

**DOI:** 10.1038/s41598-023-40263-w

**Published:** 2023-08-13

**Authors:** Wolfgang Mehringer, Maike Stoeve, Daniel Krauss, Matthias Ring, Fritz Steussloff, Moritz Güttes, Julia Zott, Bettina Hohberger, Georg Michelson, Bjoern Eskofier

**Affiliations:** 1 Machine Learning and Data Analytics Lab (MaD Lab), Department Artificial Intelligence in Biomedical Engineering (AIBE), Friedrich-Alexander-Universität Erlangen-Nürnberg (FAU), 91052 Erlangen, Bavaria Germany; 2 Department of Ophthalmology, Universitätsklinikum Erlangen, Friedrich-Alexander-Universität Erlangen-Nürnberg, 91054 Erlangen, Germany; 3 Talkingeyes & More GmbH, 91052 Erlangen, Bavaria Germany

**Keywords:** Neuroscience, Diseases, Health care, Medical research, Signs and symptoms

## Abstract

In 2019, we faced a pandemic due to the coronavirus disease (COVID-19), with millions of confirmed cases and reported deaths. Even in recovered patients, symptoms can be persistent over weeks, termed Post-COVID. In addition to common symptoms of fatigue, muscle weakness, and cognitive impairments, visual impairments have been reported. Automatic classification of COVID and Post-COVID is researched based on blood samples and radiation-based procedures, among others. However, a symptom-oriented assessment for visual impairments is still missing. Thus, we propose a Virtual Reality environment in which stereoscopic stimuli are displayed to test the patient’s stereopsis performance. While performing the visual tasks, the eyes’ gaze and pupil diameter are recorded. We collected data from 15 controls and 20 Post-COVID patients in a study. Therefrom, we extracted features of three main data groups, stereopsis performance, pupil diameter, and gaze behavior, and trained various classifiers. The Random Forest classifier achieved the best result with 71% accuracy. The recorded data support the classification result showing worse stereopsis performance and eye movement alterations in Post-COVID. There are limitations in the study design, comprising a small sample size and the use of an eye tracking system.

## Introduction

In 2019 a new Coronaviridae virus was detected called the severe acute respiratory syndrome coronavirus 2 (SARS-CoV-2), which led to a pandemic of the coronavirus disease 2019 (COVID-19)^1,2^. By the time of writing this manuscript, the total confirmed cases exceeded 760 million, and over 6.8 million deaths were reported^3,4^. Although it is called a respiratory disease, studies confirmed that multiple organs are involved in the infection’s acute phase, affording a multidisciplinary approach to the diagnosis and treatment. Symptoms include but are not limited to fatigue, muscle weakness, cognitive dysfunction, gastrointestinal symptoms, pulmonary symptoms, inflammation, etc.^5–7^.

These symptoms can persist for over 12 weeks^5,8,9^. According to recent definitions, if symptoms persist between four and 12 weeks after the acute phase of COVID-19, it is called *Ongoing symptomatic COVID-19*. If symptoms persist over 12 weeks, it is called *Post-COVID-19 syndrome* (Post-COVID)^5,8,9^. The most commonly reported symptoms in ongoing symptomatic COVID-19 are fatigue, cognitive dysfunction, and muscle weakness^10–13^. However, multiple studies showed persistent gastrointestinal symptoms, pulmonary symptoms, myocardial inflammation, and other neuropsychiatric manifestations^7,14–18^

Even in Post-COVID, fatigue, muscle weakness, and cognitive dysfunction are the main reported symptoms^8,19^. Lu et al. showed micro-structural changes in the central nervous system after 3 months leading to memory issues or smell loss, potentially also explaining the cognitive dysfunction^20^. This is supported by Voruz et al.^21^, who concluded from their study data that the nervous system is affected, and Wu et al.^22^, who observed damage to the nervous system. Other studies observed fatigue, muscle weakness, difficulties in concentration, and headaches after an extended period of 6 months^23,24^. Crivelli et al.^25^ even stated that cognitive impairments could last up to 7 months after COVID.

Accompanied by the damaged nervous system, altered eye movements^26,27^ and pupil responses^28,29^ can occur, potentially leading to reduced stereopsis performance. This highlights the importance of visual performance for Post-COVID.

Although the prevalence seems unclear, the number of patients with Post-COVID is considerable and can exert pressure on the healthcare system^30,31^. Especially the long course of Post-COVID and the accompanied limitations in daily activities might lead to societal issues^32,33^. Thus, efficiently assessing and managing Post-COVID patients is crucial. Due to multiple organs being involved and a fuzzy disease pattern, assessment and management are symptom-oriented, multidisciplinary, and a diagnosis of exclusion (i.e., exclusion of other causes of illness by multiple assessment procedures), including chest imaging, blood samples, pulmonary function tests, among many other procedures^5,9,34–36^. Therefore, several interesting approaches can be found that try to find new objective measurements for COVID, Ongoing symptomatic COVID-19, and Post-COVID for further insights into how the organism is affected but also approaches that try to classify a patient to automatically support the diagnosis of exclusion.

This paper presents a new method combining eye tracking and stereoscopic tasks to gain more knowledge about ocular functions and cognitive impairments in Post-COVID patients. Furthermore, we provide a visual and cognitive symptom-oriented assessment procedure. We used a Virtual Reality (VR) environment to show the stereoscopic visual stimuli. The stimuli vary in levels of difficulty. Accordingly, we can measure the reaction time and accuracy of responses for each difficulty and compare the results. The pupil diameter and gaze were continuously tracked while the participant responded to the stimuli to measure and evaluate the influence of stereoscopic visual stimuli on these parameters. We trained machine learning models to test the feasibility of classifying Post-COVID. Machine learning models are algorithms that can learn from data without a human having to program their behavior explicitly^37^. Thus, we provide the collected data and the so-called labels that experts created declaring the data as belonging to a control or a Post-COVID patient. Our work, therefore, contributes as follows:

- It describes a visual and cognitive symptom-oriented assessment procedure;
- It provides features resulting from the eye tracking and stereopsis performance data;
- It proposes a classification pipeline to distinguish controls from Post-COVID patients.

## Related work

In the following section, we will show and discuss related work already applying machine learning approaches to the classification of COVID. We also address results on ocular manifestations this work is based on. Furthermore, we highlight the previous work of our team that was also used in the presented approach^38–43^.

### COVID assessment using machine learning

Abunadi et al.^44^ developed a classification algorithm that automatically detects COVID in patients based on chest Computed Tomography (CT) images. They trained a deep convolutional neural network based on publicly available radiology image datasets and reached a performance of approximately 94%. Although this algorithm is designed for detecting COVID and not Post-COVID, it would be possible to monitor the changes in chest CT and use the tool for patient management. Phetsouphanh et al.^45^ observed activated innate immune cells in Long-COVID (meaning Ongoing symptomatic COVID-19) patients compared to other COVID recovered not showing persistent symptoms. Based on their findings, they built a linear classification model to differentiate between the two study populations reaching an accuracy of about 82%. They concluded that the immunological parameters could help develop prevention and treatment possibilities. These are two examples from multiple approaches applying classification or other machine learning algorithms to COVID-19. As shown by Khan et al.^46^ and Meraihi et al.^47^, CT or X-Ray images are often used for these algorithms.

Nevertheless, the examples are invasive (i.e., blood samples) or emitting radiation (i.e., CT scans). Thus, they are at least inconvenient if applied regularly to patients. Furthermore, since the current practice tends to be based on a diagnosis of exclusion, additional symptom-oriented assessment tools might be beneficial. Consequently, studies suggest further investigation to find objective measures to improve patient assessment and management for COVID, Ongoing symptomatic COVID-19, and Post-COVID^36,48,49^.

### Ocular manifestations

As indicated above, common symptoms of Post-COVID are fatigue, muscle weakness, and difficulty concentrating, but many other symptoms were also observed. For example, ocular impairments in COVID patients (often due to retinal manifestations in vein occlusions) were reported, potentially leading to visual impairment^50–57^. Brantl et al.^58^ found no significant changes in the visual functions after three months. However, they also state that the database is insufficient to be certain. Yong^59^ revealed that the brainstem is affected, and Long-COVID symptoms are persistent due to the slow regeneration of these cells. Furthermore, brainstem lesions can lead to altered eye movement behavior, in particular the peak velocity of the eyes^27^. Garcia Cena et al.^26^ also found that the time to react to a stimulus is prolonged.

Further, alterations in pupil responses were found. Bitirgen et al.^28^ found a higher latency and lower duration of pupil contraction in Long-COVID, which appears to be correlated with the symptom severity. Yurttaser Ocak et al.^29^ found that the dilation speed is reduced in COVID patients. Although Bitirgen et al.^28^ stated that the alterations could be measured in Long-COVID patients, Yurttaser Ocak et al.^29^ contradicted that the alterations are not present three months after the acute phase of COVID. Thus, the effects of COVID on ocular functions appear contentious and interesting to investigate further.

### Cognitive assessment

As stated in the “Introduction”, the difficulties in concentrating seem to originate from the involvement of the nervous system. The general term *brain fog* is used for difficulties in concentrating and fatigue. Multiple different self-report scales are available to assess brain fog in which the patients rate their state^5,60,61^. However, Lynch et al.^62^ doubt the usefulness of such scales.

In previous work, we showed methods for creating cognitively demanding purely stereoscopic visual tasks in which different difficulty levels influence participants’ reaction time^38–43^. Consequently, we hypothesize that these methods could also be used to quantify the cognitive impairment of Post-COVID patients.

Although there already exist approaches to automatically classify patients to support the diagnosis of exclusion, to the best of our knowledge, there is no work objectively assessing ocular changes and cognitive impairment. However, due to the involvement of the nervous system, we expect measurable effects on both ocular functions and cognitive performance. Therefore, these measurable effects could serve as features for classification algorithms.

## Materials and methods

In the following section, we present our approach. First, the used hardware is briefly described, followed by the created virtual environment. Furthermore, the study design and procedure are explained. Finally, the data pre-processing, feature extraction, classification pipeline, and evaluation are specified.

### VR headset

To display the VR environment, we used the HTC Vive Pro Eye (HTC Corporation, Taoyuan, Taiwan). The headset contains two 3.5″ OLED displays at a resolution of 1440 $\times$ 1600 pixels each, and a refresh rate of 90 Hz. According to the manufacturer, the field of view (FoV) is 110$^\circ$. To account for a user’s interpupillary distance (IPD), the distance between the lenses can be adjusted to 60–73 mm. The headset also contains a built-in Tobii eye tracking system (Tobii AB, Danderyd, Sweden) with a 120 Hz sampling rate and an accuracy of 0.5–1.1$^\circ$ within 20$^\circ$ FoV. In addition, the headset’s movements are tracked in 3D space using HTC’s base stations.

**Figure 1:**
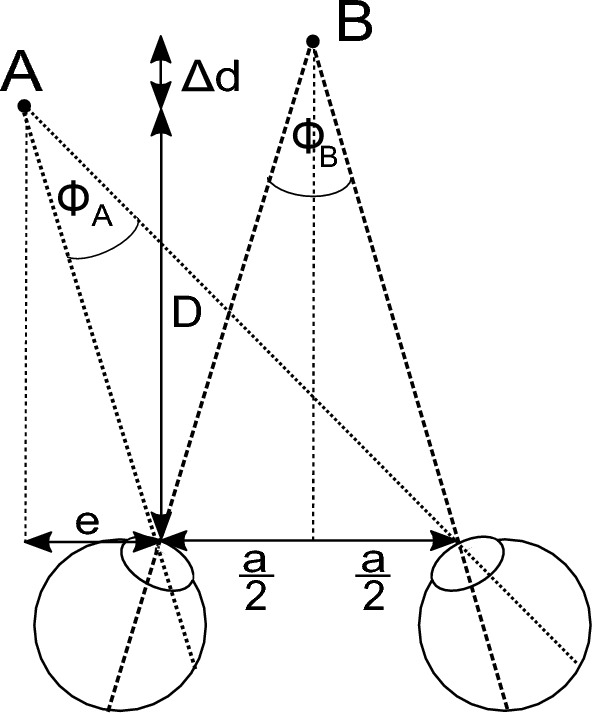
Definition of the disparity difference. The included angle between an object and the two eyes describes a disparity. Object *A* and *B* define different disparity values. The difference between those disparities defines the level of difficulty in our system. *D* defines the distance from user to object *A* and $D + \Delta d$ describes the distance from user to object *B*. Object *B* shows no horizontal shift and is half the IPD (i.e., $\frac{a}{2}$) away from each eye. Object *A* is horizontally shifted by *e*. Image based on^39^.

As mentioned, we aimed for purely stereoscopic visual stimuli with different difficulty levels. The difficulty level in a stereoscopic visual stimulus can be expressed as disparity difference^38,39^ which is shown in Fig. 1. The smaller the disparity difference, the harder it is to differentiate which ball is closer. Thus, the smaller the disparity, the better the stereopsis. If we focus on an object, the included angle between the object and the two eyes defines a disparity. If two objects are visible, two disparities can be retrieved. Subtracting the two disparities yields the disparity difference, representing the difficulty level and given in seconds of arc (arcsec or ″). Disparities are rendered by disparate image points on the projection plane^63^. Thus, the minimum disparity and the minimum disparity difference depend on the pixel spacing of the headset. Therefore, we followed the assumption made in our previous work, which is the following^39^: The horizontal resolution of 1440 pixels each display is shown across an FoV of 110$^\circ$. Consequently, the minimum disparity difference $dd_{min}$ can be expressed as

1 $$\begin{aligned} dd_{min} = \frac{110^\circ }{1440\,pixels} \cdot \frac{3600''}{1^\circ }. \end{aligned}$$

According to Eq. (1), the minimum disparity difference $dd_{min}$ is 275″ per pixel. We only allowed multiples of this value to create different difficulty levels. Richard Musil states that the FoV given by the manufacturer is too high and is, in fact, at about 107$^\circ$ which would result in approximately 268″ (see https://risa2000.github.io/hmdgdb/). Nevertheless, we decided to stick with the manufacturer’s specification since the discrepancy is subtle.

### VR environment and stimulus

**Figure 2:**
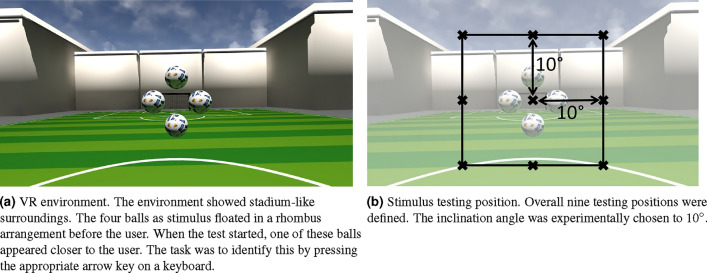
VR environment and stimulus.

We used the Unreal Engine Version 4.26 by Epic Games (Epic Games, Inc., Raleigh, USA) to create the VR environment and the stimulus. The environment consisted of stadium-like surroundings depicted in Fig. 2a. This sport-like environment was due to the previous works from Paulus et al.^42^, and Schoemann et al.^43^ that this work was based on. Furthermore, rich environments are said to be motivating. Neumann et al.^64^ showed this fact, especially emphasizing sports-related tasks.

The visual stimulus consisted of four balls in a rhombus-like arrangement shown in Fig. 2a. The balls had a distance of 200 cm from the user, and each ball was created using the standard sphere mesh with the 3D scale factor set to 0.25, resulting in a diameter of 25 cm which resembled a small football. This ball size was experimentally found convenient to look at at the given distance. The middle of the balls’ rhombus arrangement marked the center of the stimulus, which defined the overall position of the stimulus $\textbf{p}_{stim}$. This center was not visible to the user. The distance of each ball to the center was 25 cm either vertically or horizontally. Thus, the four balls were defined as $Ball_{Up}$, $Ball_{Down}$, $Ball_{Left}$, and $Ball_{Right}$. These definitions were later used to identify which ball the user was looking at.

The stimulus was tested in nine positions. Figure 2a shows the central position where the stimulus was floating directly in front of the user. Additionally, eight peripheral positions were defined by spherical coordinates. The black crosses in Fig. 2b represent all nine test positions. The radius *r* given in spherical coordinates marked the distance between the user and stimulus; as mentioned, this was 200 cm. The inclination angle $\theta$ was set to 10$^\circ$. This value was experimentally chosen since values above 10$^\circ$ cannot be displayed correctly without distortions induced by the lenses in the VR headset. We defined eight equidistant azimuth angles $\varphi \in \{0^\circ ,45^\circ ,90^\circ ,135^\circ ,180^\circ ,225^\circ ,270^\circ ,315^\circ \}$. Hence, the position $\textbf{p}_{stim}$ of the stimulus could be retrieved using the formula

2 $$\begin{aligned} \vec{p}_{stim}= r \cdot \left( \begin{array}{c} \cos {\theta } \\ \sin {\varphi } \cdot \cos {\theta } \\ - \cos {\varphi } \cdot \sin {\theta } \end{array}\right) . \end{aligned}$$

The Eq. (2) results from a differently defined coordinate system in the Unreal Engine (x-forward, y-right, z-up). Thus, the common spherical transformation equation for retrieving cartesian coordinates was rotated by 90$^\circ$ around the y-axis.

Therefore, it was possible to define nine different testing positions within the FoV of the user, namely the eight peripheral positions *Down, Lower Right, Right, Upper Right, Up, Upper Left, Left, and Lower Left* here given in the same order as $\varphi$, and the *Central* position that could also be calculated using Eq. (2) with both $\theta$ and $\varphi$ set to 0$^\circ$. To avoid losing sight of the stimulus and to ensure that the user had to shift the gaze to focus on the different stimulus positions, it followed the user’s head rotation and position so that the relative position to the user was always preserved. One of the authors patented this procedure in Europe, and a patent is pending in the US^65,66^.

As shown in Fig. 1, a disparity difference corresponds to a different distance of the objects depicted as *D* and $\Delta d$. In our case, one of the balls was closer to the user than the other three balls, which were at the initial 200 cm distance. To calculate *D* for the closer ball based on a given multiple of the minimum disparity difference $dd_{min}$, we used the following assumptions that can be found in Fig. 1: The disparity difference $\Theta$ is the difference between the two disparities $\Phi _{A}$ and $\Phi _{B}$ and was set to a multiple of $dd_{min}$.

3 $$\begin{aligned} \Theta = \Phi _{A} - \Phi _{B}. \end{aligned}$$

$\Phi _{B}$ is known since $D + \Delta d$ was given (i.e., 200 cm), and according to Jan Paulus^67^, we could set the IPD to the mean human IPD of 6.3 cm because the observer distance $D + \Delta d$ is significantly larger than the IPD. Thus, the small interindividual changes in the IPD will have a negligible effect. Hence, we calculated $\Phi _{B}$ using

4 $$\begin{aligned} \Phi _{B} = 2 \cdot \tan ^{-1}{\left( \frac{a}{2 \cdot (D + \Delta d)} \right) }, \end{aligned}$$

where *a* denotes the IPD in accordance with Fig. 1. Since $\Phi _{A}$ is shifted horizontally by *e*, we accounted for that by defining the distance of object *A* to each eye by

5 $$\begin{aligned} l_{1} = |e|,\hspace{0.5cm} l_{2} = |e| + |a|. \end{aligned}$$

$\Phi _{A}$ could be calculated by using

6 $$\begin{aligned} \Phi _{A} = \tan ^{-1}{\left( \frac{l_2}{D} \right) } - \tan ^{-1}{\left( \frac{l_1}{D} \right) }. \end{aligned}$$

Since $\Phi _{A}$ is a subtraction of two angles, we could apply the addition theorem of the tangent. By reformulating Eq. (3) and multiplying both sides with the tangent, we receive

7 $$\begin{aligned} \tan {\left( \Theta + \Phi _B \right) } = \frac{\frac{l_2 - l_1}{D}}{\frac{D^2 + l_1 \cdot l_2}{D^2}}. \end{aligned}$$

For simplification, we substituted the left-hand side of Eq. (7) with $c_1$ as constant and we also reformulated this equation to obtain

8 $$\begin{aligned} c_1 \cdot D^2 + (l_1 - l_2) \cdot D + c_1 \cdot l_1 \cdot l_2 = 0. \end{aligned}$$

We defined two more constants $c_2$ and $c_3$ (see Eq. 9) to finally obtain the quadratic equation shown in Eq. (10).

9 $$\begin{aligned} c_2 = l_1 - l_2,\hspace{0.5cm} c_3 = c_1 \cdot l_1 \cdot l_2, \end{aligned}$$

10 $$\begin{aligned} c_1 \cdot D^2 + c_2 \cdot D + c_3 = 0. \end{aligned}$$

We could now solve the quadratic equation for D.

Since solving this quadratic equation resulted in two values for *D*, we checked whether the result was smaller than the initial $D + \Delta d$ (i.e., 200 cm) or larger and only kept *D* that was smaller. We applied this formula to calculate *D* for each of the nine testing positions.

As described in our previous work, a positional change of an object will result in monocular depth cues due to the *size of known objects* and the *linear perspective*^39^. To account for that, we applied the same correction to the size of known objects by scaling down the closer ball by the factor *s* (see Eq. 11), which was applied to the 3D scale of the ball’s mesh with

11 $$\begin{aligned} s = \frac{D}{D + \Delta d}. \end{aligned}$$

The linear perspective was solved by adding a random variation to each ball. Each ball received a random variation between 0 and 2 cm (i.e., empirically evaluated) each time a new stimulus was created. This variation was horizontally added to or subtracted from the balls’ position of $Ball_{Left}$ and $Ball_{Right}$ and vertically added to or subtracted from the balls’ position of $Ball_{Up}$ and $Ball_{Down}$. This random variation veiled the effect of linear perspective by creating different views for each ball.

The remaining monocular depth cues listed in our previous work were addressed in the following way^39^: The cue *overlay of contours* was canceled out by avoiding overlaps in the scene. The cue *distribution of highlights and shadows* was avoided by not casting shadows and using one light source that was far away (i.e., the sun in the sky sphere). The *aerial perspective* was not rendered in the scene. Finally, the *motion parallax* was avoided by attaching the stimulus to the user’s head rotation and position so that the relative position to the user was always preserved.

### Study design and procedure

The study 2490-PC-2021-V14 ”*Discover-Establishment and evaluation of clinical algorithms for objective diagnosis of subtypes of Long-COVID as an essential basis for effective patient care*” was designed as a prospective case-control study at the Department of Ophthalmology, University of Erlangen-Nürnberg, Friedrich-Alexander-Universität Erlangen-Nürnberg (FAU). Thirty-five subjects were recruited for this pilot study: 20 patients with Post-COVID (age: 29±4 years, gender eight female, 12 male) and 15 controls (age: 25±2 years, gender eight female, seven male). Post-COVID syndrome is defined as persistence of symptoms $\ge$12 weeks after SARS-CoV-2 infection to the S1 Guideline^9^. SARS-CoV-2 infection was confirmed by a positive real-time reverse transcription polymerase chain reaction test. Persistency of Post-COVID symptoms was 389.25±189.34 days at recruitment. Anamnestic data of self-reported Post-COVID symptoms were recorded. The patients underwent an ophthalmic examination (e.g., measurement of best-corrected visual acuity (BCVA), non-contact intraocular pressure (IOP)) next to serological and internal examinations. Best-corrected visual acuity was 1.0±0.0 (Post-COVID patients) and 1.0±0.0 (controls). The spherical equivalent was −0.12±0.49 (Post-COVID patients) and −0.62±1.92 (controls). The most common self-reported Post-COVID symptoms in the present cohort were post-exertional malaise (100%), muscular pain (100%), headache (100%), and a Bell Score of 36.50±10.27. The Bell Score defines the disability extent caused by chronic fatigue syndrome by giving points ranging from 100 to zero, where 100 means no symptoms and zero symbolizes a patient requiring assistance to get out of bed^68^. No local or systemic disorders with retinal affection were recorded within this cohort. The study was approved by the Ethics Committee of the Friedrich-Alexander-Universität Erlangen-Nürnberg (see https://www.ethikkommission.fau.de/, protocol code: 295_20 B), and written informed consent was obtained from all participants involved in the study following the declaration of Helsinki. We confirm that all methods were performed in accordance with the relevant guidelines and regulations.

Before starting the VR program, the participants were all introduced to the VR headset. Thereon, the VR environment and the stimulus were explained using similar images like Fig. 2a. The participants were instructed to use the arrow keys on a wired keyboard to decide which ball was closer. Thus, the keyboard was also shown and placed in front of them on a desk.

Participants with spectacles were asked to participate without spectacles since this might influence the accuracy of the eye tracking system. During the first trial (see below), we only asked if the image was sharp enough to see the balls and identify a difference. Therefore, we considered their performance as a confirmation of their statement.

After the pre-tests, the participants were advised to wear and familiarize themselves with the headset. We used a PC with an AMD Ryzen 9 CPU, 32 GB RAM, and RTX2070 graphics card. Subsequently, HTC’s SRanipal runtime (version 1.3.1.1) was used to perform a 5-point calibration for the eye tracking system. The calibration procedure additionally checked the IPD settings and headset positioning on the head to achieve optimal headset configuration and positioning.

After finishing the calibration, each participant completed three trials of the test paradigm. At the beginning of each trial, the four target balls were on the same level, so no disparity difference was set. This was entitled the *idle* phase. The participant could start the trial by pressing one of the arrow keys. Each trial consisted of 81 stereoscopic visual stimuli (explained in “VR environment and stimulus” section) resulting from three tested disparities $\in \{275, 550, 1100\}$, the nine testing positions, and three repetitions of the combinations of each disparity and testing position. The order of the 81 stereoscopic tasks was random. In each trial, the participant was to identify the closer ball and press the appropriate arrow key on the keyboard as fast as possible. The first trial was performed to familiarize the participants with the task and program. The second trial was considered training the system so that learning effects could be avoided. Then, the data from the third trial were considered for further processing. Suppose a low performance close to random guessing occurred (i.e., 25% of stimuli correctly identified) in the first and second trials. In that case, the participant could have been excluded from the study as unable to perceive the depth information. This did not occur in our study population.

We intentionally decided on this number of disparities, stimulus repetitions, and testing positions because patients already underwent multiple assessments taking part in the study. Thus, we did not want to overburden the participants but provide them with a proper familiarization and training phase. This is why we repeated the whole test three times. Moreover, we intentionally wanted to test the system’s sensitivity to variability in the data based on a short testing time. The disparities were chosen based on the two most difficult, resulting in 275″ and 550″. The additional choice of 1100″ was also to test an easier disparity.

For each trial, we recorded 81 responses to the stereoscopic visual stimuli. Each response consisted of the reaction time (i.e., time from stimulus onset until an arrow key was pressed), correctness (i.e., whether the closer ball was correctly identified), the disparity setting, and the testing position. While performing the stereoscopic visual stimuli, the pupil diameter, the gaze direction, and the gaze origin for both eyes were recorded. Therefore, we used HTC’s SRanipal plugin for Unreal (version 1.3.3.0). The eye tracking data were synchronously sampled with the frame rate of the VR environment’s rendering (tick event in the Unreal-Engine). The pupil diameter was recorded in millimeters for the left and right eye. In addition, gaze direction and gaze origin were recorded as 3D vectors. The origin defines the position of the left or right eye in the VR environment. The gaze direction describes a normalized vector following the gaze of the left or right eye inside the VR environment. Based on the gaze direction, the fixated ball was extracted so that for each sampling point $Ball_{Up}$, $Ball_{Down}$, $Ball_{Left}$, $Ball_{Right}$ or (in case non of the balls were fixated) *nan* was recorded. Furthermore, the SRanipal plugin provides an eye openness value for the left and right eye where 1 denoted an open eye and 0 a closed eye. Hence, the data consisted of three groups, namely *stereopsis performance*, *pupil diameter*, and *gaze behavior*.

Therefore, this system measures the reaction time and accuracy over different difficulty levels in the stereoscopic visual stimuli (i.e., the different disparities). It also measures the pupil response and gaze over different difficulty levels. Accordingly, we evaluate how the two groups perform at each disparity and how they perform across the disparities to compare the groups’ results. Additionally, we evaluate how the pupil response and gaze change across the different disparities and groups. We do not measure and evaluate the best stereoacuity.

**Figure 3:**
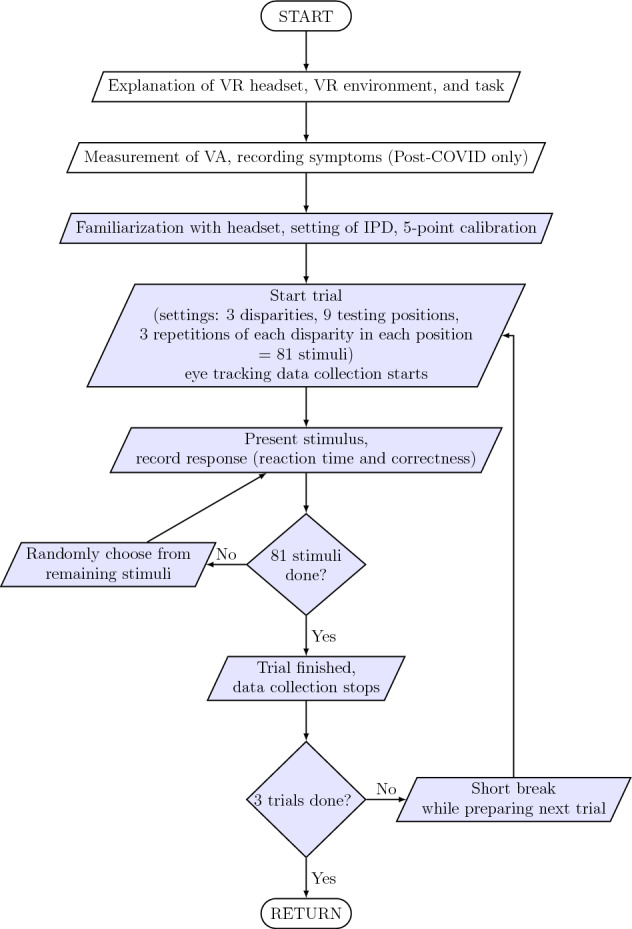
Flowchart of the study procedure. The light blue parts of the flowchart represent the steps in which the participant was within the VR environment and simulation.

For better visualization and ease of understanding, the flowchart of the study design is shown in Fig. 3.

### Data pre-processing

To extract the *pupil diameter* and *gaze behavior* data, we performed pre-processing to account for noise and artifacts (due to blinking). Furthermore, we adapted the pipeline by Stoeve et al.^69^ based on the pipeline of Kret and Sjak-Shie^70^ to enhance the signal quality of the *pupil diameter* data. We excluded all blinks and non-valid samples from both data groups. The blinks were identified using the *eye openness* parameter. If the value was below 0.1, the sample was marked as a blink, according to Alsaeedi et al.^71^. Since we did not further process the information on blinks, we retained their initial threshold of 0.1.

#### Pupil diameter data

For pre-processing the *pupil diameter* data, we applied the filtering techniques explained by Kret and Sjak-Shie^70^. The following brief list highlights the five raw pupil diameter data filtering steps^70^:

- Pupil diameters that were outside a specified range were rejected. The range defines the physiological behavior of pupils and spans from 1.5 to 9.0 mm.
- Pupil diameters with substantial changes between subsequent samples were rejected. These changes can occur due to blinks and other artifacts.
- Pupil diameter samples far off a fitted trend line were rejected.
- Small clusters of pupil diameter samples that were temporally isolated were rejected.
- The mean pupil diameter data were calculated from the left and right eye, and missing data were interpolated.

**Figure 4:**
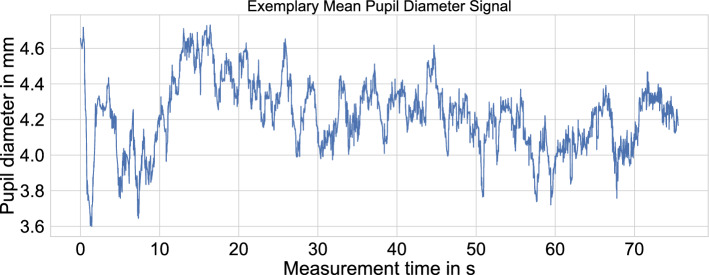
Exemplary mean pupil diameter signal after applying the filtering steps explained by Kret and Sjak-Shie^70^.

As part of the last step, Kret and Sjak-Shie used a linear interpolation technique in case of missing pupil diameter data from both pupils due to previous filtering steps. However, we followed the implementation by Stoeve et al.^69^, who applied the Piecewise Cubic Hermite Interpolating Polynomial (Pchip) method due to results from Dan et al.^72^ investigating the fit of various interpolation methods. For further information on the individual steps and the implementation, Kret and Sjak-Shie published their code at https://github.com/ElioS-S/pupil-size. An exemplary mean pupil diameter signal for one participant is given in Fig. 4.

#### Gaze behavior data

For the *gaze behavior* data, we applied a linear interpolation method to fill the missing data due to the removed blinks and invalid data samples. We decided on linear interpolation because the missing data gaps were small (i.e., 10 sample points matching approximately 220 ms). Thus, the gaze change was expected to be directed and follows a linear path.

### Feature extraction

In total, we extracted 73 features from the three different data groups. Regarding machine learning, features are the input for the model^73^. In our case, the goal of the model is to learn how to differentiate between control and Post-COVID by finding a pattern in the input features. We are oriented on existing literature that extracts and creates similar or comparable features^69,74–77^. In the following, the features are explained in more detail. In addition, as part of the supplementary material, Figs. 1, 2, and 3 show visualizations of all normalized features.

#### Stereopsis performance features

For the *stereopsis performance* data, we extracted 29 features resulting from nine features for each of the three disparities and two additional features. We only evaluated the correct responses from the 81 stimuli responses of each run. For example, if a participant identified 70 balls correctly, we only evaluated the reaction time and accuracy of those 70 balls. Based on the remaining data, we extracted the *maximum, minimum, mean, median, standard deviation, variance, skewness, and kurtosis* of the reaction time for each disparity. Additionally, we calculated the *accuracy*, which defines the number of correctly identified balls divided by the total number of balls tested in the respective disparity. The two additional features were calculated by subtracting the median reaction time of two disparity settings. The resulting difference was called the gain and is described in^65^ as the difference between the easiest disparity setting and a more demanding setting. In that way, the individual base response time can be eliminated, and only the reaction time difference between easy and more difficult tasks can be evaluated. Hence, we calculated the gain between 275″ and 1100″ and between 550″ and 1100″.

#### Pupil diameter features

Eighteen features were extracted for the *pupil diameter* data. We extracted eight statistical features *maximum, minimum, mean, median, standard deviation, variance, skewness, and kurtosis* based on the mean pupil diameter signal of the complete test run (i.e., over the complete sequence of 81 stimuli). Furthermore, we calculated four features using the *Low/High Index of Pupillary Activity (LHIPA)* as performed by Duchowski et al.^78^. The LHIPA measures the cognitive load based on the oscillation and variation in the pupil diameter signal. The more stable the signal, the less cognitive load is expected, and the more variable (i.e., oscillating) the signal, the higher the cognitive load^78^. The ratio between high and low-frequency bands of the pupil diameter signal can be calculated using wavelet decomposition. Thus the pupil response to different cognitively demanding tasks can be compared. In our case, the cognitively demanding tasks are the different disparity settings. Note that a lower LHIPA value represents more oscillation in the data and, therefore, a higher cognitive load. We computed a baseline LHIPA based on one second before a disparity was set (i.e., during the *idle* phase). Moreover, LHIPA values were calculated for each disparity. Since the order of the stimuli was random, the mean pupil diameter signal was subdivided based on the 81 stimuli. Finally, the LHIPA value was calculated and averaged across each subdivision with the same disparity. We divided the 81 subdivisions into two parts and calculated the signal slopes using linear regression as presented by Baltaci et al.^79^. The signal was divided because the pupil response to the stimulus is delayed^80–83^. So the first part of each subdivision might have been corrupted by the pupil’s response to the preceding stimulus. The second part better represented the pupil’s response to the current stimulus. The two resulting slopes for the 81 subdivisions were averaged over each disparity. Hence, we received another six features.

#### Gaze behavior features

From the *gaze behavior* data, we extracted 26 features. We chose the asymptotic model described by Duchowski et al.^84^. The proposed model calculates the angular velocity based on the angular change in each frame. In our case, we sample the gaze direction (i.e., normalized 3D vector where the person was looking at in the VR environment) at 90 Hz. Thus, we receive the gaze direction each 11 ms. Subsequently, the angular change can be calculated using the dot product of two consecutive gaze directions. Now, the asymptotic model is applied to calculate the angular velocity, and a threshold is set below which a fixation is detected. We applied the algorithm to the data of each eye so that the head position mentioned by Duchowski was the gaze origin of the respective eye.

The resulting *number of fixations* for the right and left eye were added as features. Additionally, we extracted the seven statistical features *maximum, mean, median, standard deviation, variance, skewness, and kurtosis* for the angular velocity based on the asymptotic model. The minimum angular velocity was zero for each participant in both Post-COVID patients and controls. Consequently, we did not consider the minimum angular velocity as a feature. As mentioned in *VR Environment and Stimulus*, whether the participant focused on a specific ball or in the background was recorded. The time spent on each area was also used as a feature.

### Classification pipeline

We adapted the classification pipeline by Stoeve et al.^69^ by comparing the performance of various classifiers. We chose the following classifiers: Support Vector Machine (SVM) with the linear kernel as well as radial basis function (RBF) kernel^85^, Random Forest (RF)^86^, and K-Nearest Neighbors (kNN)^87^. We performed a grid search to find the optimal parameter settings for the model (i.e., hyperparameter) with similar ranges described by Stoeve et al.^69^. However, we chose a variation in the hyperparameter *Maximum depth* for the RF classifier. Furthermore, the RF parameters *n_estimators, max_features, and bootstrap* were set to $\{300, auto, True\}$ respectively. The parameter search space is summarized in Table 1. To ensure comparable results and reproducibility, we set the *random_state* (if available for the classifier model) to *zero*. The features were normalized based on the training data to a range between zero and one using the *MinMaxScaler* from the sklearn python package^88^.

**Table 1:** Parameter search space for each classifier.

Classifier	Hyperparameter	Search space
SVM (lin kernel)	Cost parameter (c)	$2^N, N \in \{-10, -9,..., 10\}$
SVM (rbf kernel)	Cost parameter (c) gamma	$2^N, N \in \{-10, -9,..., 10\}$ $2^N, N \in \{-10, -9,..., 10\}$
kNN	# of neighbors weights	$\in \{3, 4,..., 15\}$ $\in \{uniform, distance\}$
RF	Maximum depth Min. # of samples to split Min. # of samples at leaf n_estimators max_features bootstrap	$\in \{2, 4, 6\}$ $\in \{2, 4, 6\}$ $\in \{1, 2, 4\}$ $\in \{300\}$ $\in \{auto\}$ $\in \{true\}$

Based on Stoeve et al.^69^.

As proposed by Stoeve et al.^69^, we applied nested cross-validation (NCV) in which the inner loop consisted of a fivefold Cross-Validation (CV) for parameter tuning across the search space. A CV splits the data into the requested number of folds. For example, a fivefold CV splits the data into five groups based on the participants. The data were split using the StratifiedKFold function from the *sklearn* python package to ensure the same class distribution in the resulting splits^88^. The class here represents the individual participant as belonging to control or Post-COVID. We set the StratifiedKFold’s parameters *shuffle* and *random_state* to *true* and *zero*, respectively. We applied a feature selection to evaluate each feature’s importance. However, we did not use the selection to optimize for the best number of features since we were interested in testing all available features. Moreover, we manually performed a feature selection by subdividing the data based on the three data groups. Additionally, computational complexity was not considered a critical factor.

The parameter combination that achieved the inner CV’s best average performance was chosen and applied to the outer CV. For the outer CV, we applied a Leave-One-Out Cross-Validation (LOOCV) to evaluate the optimized model performance for each participant. The LOOCV leads to the least biased performance estimation and should be applied when working with clinical data since it mimics the clinical use-case scenario of diagnosis^89^. As stated by Stoeve et al.^69^, the pipeline was used for each classifier to ensure the comparability of classification results.

### Model evaluation

To evaluate the performance of the individual data groups, we used only the particular feature subsets as input for the classification pipeline but also a combination of all subsets. The result of the feature selection was summarized by providing the top ten features, including all data groups. The top ten represent the best features for differentiating between control and Post-COVID. We identified the best parameter settings based on the fivefold inner loop of the NCV. Only the three most frequent parameter settings were reported and summarized.

Since we applied a LOOCV, we evaluated a single participant in the outer loop of the NCV. However, each participant only has one sample (i.e., one value) for each feature. Thus, the accuracy for this participant is either zero (e.g., the participant belongs to control but was classified Post-COVID) or one (e.g., the participant belongs to control and was classified control) for the overall model performance (outer loop of NCV). Hence, the mean accuracy across all 35 participants is given in steps of approximately 2.9% (i.e., 100% if all participants were classified correctly, and 97.1% if only one participant was misclassified). Likewise, the standard deviation was large due to either zero or one accuracy. Therefore, we did not report the standard deviation. Instead, we created confusion matrices for the best-performing classifier in which the number of correctly and incorrectly classified participants are shown.

## Results

In the following, the results of our study are shown. The chapter is subdivided into two parts according to the contributions listed in the “Introduction”. Thus, the first part is based on providing suitable features resulting from eye tracking and stereoscopic visual stimuli. The second part is based on proposing a classification pipeline to distinguish controls from Post-COVID patients.

### Feature rating

The top ten features from the feature selector were identified to be the following: the fixation duration on the background for the right eye (nan_Fixation_Duration_Right *(Gaze)*), the number of fixations done by the left and right eye (Number_Fixations_Left & Number_Fixations_Right *(Gaze)*), the mean reaction time for the disparity 1100″ (Mean_1100.0 *(Stereo)*), the kurtosis of the reaction times for the disparity 275″ (Kurtosis_275.0 *(Stereo)*), the minimum reaction time for the disparity 275″ (Minimum_275.0 *(Stereo)*), the fixation duration on the background for the left eye (nan_Fixation_Duration_Left *(Gaze)*), the kurtosis of the right eye’s angular velocity based on the asymptotic model (Kurtosis_Right *(Gaze)*), the median reaction time for the disparity 1100″ (Median_1100.0 *(Stereo)*), the kurtosis of the left eye’s angular velocity based on the asymptotic model (Kurtosis_Left *(Gaze)*).

The result of the classification run combining all feature categories was evaluated to investigate the overall performance. The main contributions in the top ten features were provided by *stereopsis performance* and *gaze behavior* data indicated by *Stereo* and *Gaze* in the listing above. Specifically, the fixation duration and the number of left and right eye fixations were valuable features from the *gaze behavior* data. In addition, from the *stereopsis performance* data, the reaction time’s minimum, mean, and median were chosen. However, no features from the *pupil diameter* data were selected.

### Classification results

Before looking at the classification accuracy, the resulting parameter settings of the inner CV are shown. The best parameter settings for each classifier and each data group are given in Table 2. The numbers in brackets written in italic behind the respective parameter settings show the number of times this setting was selected in the inner CV. Overall, it can be seen that none of the parameters was solely selected from the extremes of the search space. Instead, the parameters are drawn from a wide range of the search space.

**Table 2:** The three best parameter settings for each classifier and each data group based on the fivefold inner CV.

Classifier	Parameter names	Stereopsis performance	Pupil diameter	Gaze behavior	All
SVM (lin kernel)	c	0.063 *(9)* 0.001 *(8)* 0.125 *(5)*	0.001 *(13)* 1.000 *(8)* 256.000 *(5)*	0.250 *(12)* 0.001 *(7)* 1.000 *(7)*	0.063 *(9)* 0.001 *(8)* 1.000 *(6)*
SVM (rbf kernel)	c, gamma	1.000, 0.500 *(5)* 4.000, 0.500 *(5)* 0.500, 0.125 *(4)*	1.000, 2.000 *(10)* 1.000, 1.000 *(5)* 256.000, 0.125 *(2)*	1.000, 4.000 *(13)* 0.500, 2.000 *(8)* 0.500, 1.000 *(6)*	2.000, 1.000 *(12)* 1.000, 0.250 *(7)* 4.000, 0.250 *(4)*
kNN	n_neighbors, weights	5, uni *(9)* 11, uni *(5)* 9, uni *(4)*	5, uni *(12)* 4, dist *(6)* 3, uni *(3)*	4, dist *(8)* 3, uni *(5)* 6, dist *(4)*	7, uni *(12)* 5, uni *(7)* 10, dist *(5)*
RF	max_depth, min_samples_split, min_samples_leaf	2, 2, 2 *(11)* 2, 2, 1 *(10)* 2, 2, 4 *(10)*	2, 2, 1 *(9)* 2, 6, 1 *(6)* 2, 2, 2 *(5)*	2, 2, 4 *(15)* 2, 2, 1 *(9)* 2, 2, 2 *(2)*	2, 2, 1 *(17)* 2, 2, 2 *(6)* 2, 2, 4 *(3)*

The optimized parameter names are given. The number in brackets, written in italic, refers to the number of times this setting achieved the best average performance. The parameters were rounded to equalize decimals (e.g., 0.001 refers to rounded $2^{-10}$).

Table 3 summarizes the mean accuracies across all participants and the data groups. For the *stereopsis performance* data, the RF classifier performed best with a mean accuracy of 71%. For the *pupil diameter* data, the kNN classifier achieved the best performance reaching 49%. This is close to random guessing. The SVM classifier with rbf kernel yielded the best performance for the *gaze behavior* data with 66%. Combining all data groups, the RF classifier achieved the best accuracy with 66%. Thus, the RF classifier reached top performance in two of four cases. The SVM classifier with linear kernel performed worst. Interestingly the SVM with linear kernel performed below random guessing in each data group.

**Table 3:** Mean accuracy and standard deviation of the different classifiers across all participants and the data groups.

Classifier	Accuracy stereopsis performance (%)	Accuracy pupil diameter (%)	Accuracy gaze behavior (%)	Accuracy all (%)
SVM (lin kernel)	46	37	43	34
SVM (rbf kernel)	54	37	**66**	63
kNN	60	**49**	40	51
RF	**71**	46	60	**66**

The best mean accuracy score of the respective data group is written in bold.

**Figure 5:**
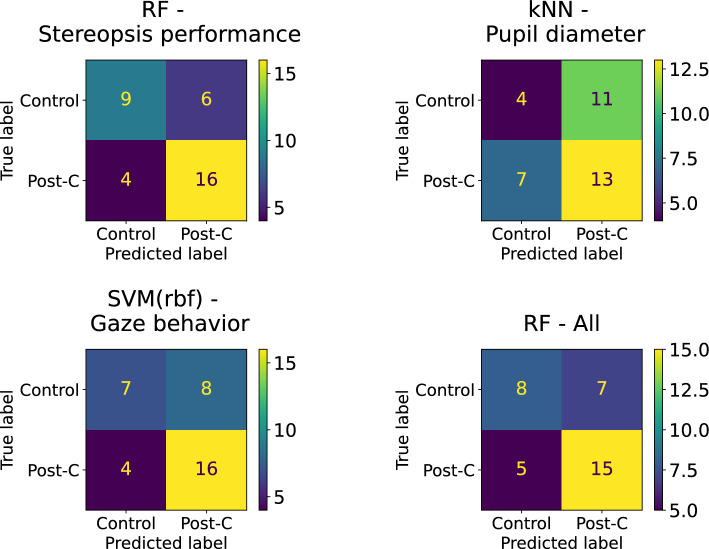
Confusion matrices for the best-performing classifiers of each data group. The rows indicate the true label, meaning the group a participant belongs to (i.e., control or Post-COVID (Post-C)). The columns represent the predicted value, meaning the classifier’s prediction of which group the participant could belong to.

The mean accuracy cannot reveal each class’s false positive and false negative rates (i.e., control or Post-COVID). Thus, Fig. 5 illustrates the confusion matrices for each data group and its best-performing classifier. Overall, it can be seen that controls were misclassified more frequently than Post-COVID patients. For example, the sensitivity in classifying Post-COVID using *stereopsis performance* data was 80% (16 out of 20). However, the specificity in classifying controls was 60% (nine out of 15).

## Discussion

The classification performance demonstrates that particularly in *stereopsis performance* and *gaze behavior* data differences between the groups are present. Classification algorithms seem to be a suitable method for highlighting these. The process of data collection only takes approximately one minute. A complete testing paradigm (three repetitions), as tested in this study with Post-COVID patients, takes 5 minutes with resting breaks in between. In the following, we will discuss our findings in more detail and propose further steps.

The features from the *stereopsis performance* data were often chosen as the most valuable. For example, if we look at the data in Fig. 1 in the supplementary material, one can see that the reaction time was increased. All three disparities showed this effect in the minimum, maximum, median, and mean reaction times. Furthermore, the standard deviation and variance were slightly increased in Post-COVID patients, indicating that some patients had a larger difference in their reaction time. Due to that, the kurtosis tended to be larger for controls. Although we could see differences, the classes were not well segregated. The overlapping boxplots in Fig. 1 indicate this in the supplementary material. Thus, some patients’ reaction time seemed not to be affected. This might explain the misclassification of Post-COVID patients and controls. Still, the reaction time measures were the top features of the classification pipeline. The accuracy was decreased for each disparity in patients with Post-COVID supporting studies from literature^11,19,21^. The neurophysiological deficits and cognitive impairments were expected to negatively influence the task’s reaction time and accuracy. However, accuracy was not selected as a valuable feature. The reason might be that one patient performed worse than the others (35% accuracy if not normalized). The remaining patients showed a lower accuracy, but no substantial difference from the controls could be observed. Therefore, the accuracy seems to be less valuable than the reaction time. Since we only evaluated correct responses for the reaction time measures, the worse performance of the mentioned patient had less impact. We decided not to exclude this patient from the dataset since the performance was above random guessing, and the pre-tests indicated visual capability to perform well. A possible reason for the worse performance could be interindividual variability so that the patient was, by chance, worse than the other. It could also be that the patient did not communicate that the task was misunderstood.

We expected a difference in the eye movements based on the asymptotic model from the *gaze behavior* data. More precisely, we expected a decrease in the velocity for Post-COVID patients because of fatigue and altered muscle function that could have led to reduced eye movement^27,59,90,91^. The results show a decrease in the velocity. This effect can be seen in Fig. 3 in the supplementary material for the median and mean on both the left and right eye. However, the maximum velocity for the right and left eye shows a much broader distribution, not clearly indicating an increase or decrease. In addition, standard deviation and variance did not differ greatly, suggesting that patients and controls might respond similarly. Skewness and kurtosis were larger in Post-COVID, indicating a data distribution towards higher velocity values. When looking at each target’s fixation duration, it can be stated that Post-COVID patients tended to take longer for the testing procedure. This coincides with the observation of prolonged reaction times. Note that the overall testing time and time spent on each target ball were increased. We assume that Post-COVID patients struggle to identify the correct ball and take more time while controls take less time to identify the closer ball. Hence, the target’s fixation durations are all increased in Post-COVID patients.

Based on the selected features, the *pupil diameter* data were considered unimportant. This indicates small or negligible differences in the data. Figure 2 in the supplementary material shows that data. Except for the LHIPA value of 275″, no features showed a great difference between Post-COVID and controls. Their alleged unimportance might result from two effects compensating for each other. On the one hand, the cognitive impairments in Post-COVID patients were expected to decrease the LHIPA value (the lower the LHIPA value, the higher the cognitive load^78^). On the other hand, the pupil response after COVID is delayed or slowed down^28,29^. Since the LHIPA value summarizes the oscillation of the pupil diameter signal, a delayed or slowed-down pupil response will increase the LHIPA value by inhibiting the high-frequency oscillations of the signal. Accordingly, there might be compensating effects mitigating differences between the two groups. However, the only feature clearly showing differences is the LHIPA under 275″. A reason could be that the cognitive impairments in Post-COVID fully come into play for the hardest stimulus we can test (275″ being the most difficult task). Thus, we can observe a decrease in the LHIPA value as expected for this disparity setting.

Furthermore, we expected to observe differences in the pupillary signal’s slope for each tested stimulus. Therefore, we divided these signals into two parts, slope 1 and slope 2. Due to a delay in response to the stimulus onset, slope 1 might be corrupted by the response to the preceding stimulus. In contrast, slope 2 represents the response to the current stimulus^79,80,82^. We expected smaller slopes for Post-COVID due to the aforementioned slowed-down response^28,29^. We found this effect in 275″ and 1100″. However, the difference was not substantial. Note that 0.5 for the slope features in Fig. 2 in the supplementary material represents 0 if not normalized. Consequently, values below 0.5 indicate a negative slope. One reason for the missing difference might be the time spent on the stimulus. Instead of showing the stimulus only a pre-defined time, the participants could spend as long as necessary before pressing an arrow key. Thus, the signal parts were too long, and subsequent pupil fluctuations distorted the response of interest. To improve this, the game must be adapted to present the stimulus only for a pre-defined time (e.g., one second).

The best parameter settings for the machine learning classification models summarized in Table 2 show that the search space seems to be well selected since no extreme value was chosen over all cases except for the maximum depth of the RF classifier (i.e., $max\_depth = 2$). However, since this RF classifier parameter is a maximum value, the true chosen value could also be one^88^. Therefore, a further reduction of the parameter was not intended, although the true prevalence of the depth being one is not known. Furthermore, since no parameter settings were consistently selected in the data groups, the performance seems robust against minor changes in the settings.

Although the search space seems well-defined, the classification performance just reached 71%. As stated above, this could arise from the data. However, another reason might be that all available features were included in the classification task. Especially for the *gaze behavior* data, we introduce a dependence on the different eyes. Each feature in this data group is calculated for the left and the right eye. Thus, they are highly related, and we might have affected the performance due to the choices of features we passed to the classification algorithm^92^.

Hence, the choice of features influences the classification performance. This also applies to the number of features. A high number of features is not always beneficial and might be solved by applying dimensionality reduction techniques^93^. Since we did not apply these, future work should investigate if such a technique can further increase the classification performance by canceling redundancy and noise introduced by the number of features^94^. Furthermore, features of other modalities could be used. For example, instead of descriptive statistics or derived features that represent a whole time series in one value, it might be interesting to apply time series analysis which also considers the changes of signals over time^95,96^. Consequently, different and potentially valuable features that replace others not beneficial for the current classification pipeline can be created.

Eye tracking in VR headsets is prone to different results for both eyes due to the short distance between the eyes and the eye tracking system. The eye tracking system must be calibrated to ensure that the placement of the cameras is optimal^97^. Still, subtle changes in the positioning of the headset on the head or the individual size of the head can lead to altered results, although the calibration might be a success^98^. Looking at Fig. 3 in the supplementary material, one can see that the data from both eyes in Post-COVID patients differed. Although the calibration was completed successfully, this could hint at a systematic false headset positioning. A mean gaze value similar to the approach described by Kret et al.^70^ could be applied to overcome the eyes’ dependence and the headset’s systematic false positioning. The mean gaze value would compensate for the systematic error and condense the two eyes. Thus, the tendency of the gaze behavior can be compared. This approach, however, should not be used if differences in gaze behavior are expected (i.e., onesided eye muscle dysfunction).

We have tested younger adults in a specific age band of 20–35 years. In older adults, however, reduced reaction times^99^, reduced stereopsis^100^, and different technology usage^101^ is reported. Although some studies revealed an age-related risk of Post-COVID^102^, other literature does not support this^103^. Accordingly, to avoid age-related confounding factors regarding stereopsis performance, we decided on this specific age band leading to the presented small dataset of 20 Post-COVID and 15 controls.

However, more data for every age and both groups Post-COVID and controls are required to confirm the findings. The need for more data is a significant limitation of this study. Therefore, it is crucial to collect balanced and larger datasets comprising comparable group members equally distributed over age bands, gender, and disease pattern. First to support and verify our findings on a larger scale and second, to test whether one model can robustly classify Post-COVID patients or if multiple models need to be trained to handle different characteristics (i.e., gender or age) separately.

Moreover, a robust classification also means the model can correctly classify data not used for training and testing. Thus, the classification should also work for new or so-called *unseen* data. In future work, unseen data should be used to test the classification accuracy outside training and testing datasets to investigate the model’s robustness.

A study limitation is introduced by testing the participants without their spectacles, thus, without the best-corrected vision. However, stereopsis relies on proper vision and visual acuity^104^. A decrease in visual acuity also decreases stereoacuity, defined as the smallest differentiable disparity^104^. This might have influenced the *stereopsis performance* data. Hence, in favor of high-quality eye tracking data, we asked the participants to take off their spectacles at the risk of corrupting the *stereopsis performance* data. A possible solution would have been to perform the test twice with and without the spectacles and take the stereopsis performance data from the run with spectacles and the eye tracking data from the run without the spectacles. Therefore, the study design incorporates only one additional run.

Another study limitation is that we did not measure the stereoacuity. Instead, we used the patients’ performance in trials one (i.e., familiarization) and two (i.e., training) to indicate sufficient stereopsis. This was done since the tested disparities were larger than adequate stereoacuity, which might be around 30″^104^. Nevertheless, without knowing the stereoacuity of individuals, we cannot estimate the influence on our measurement. Therefore, future studies should measure the stereoacuity to observe possible differences, especially in the stereopsis performance data. These changes might occur even if the stereoacuity of a patient is lower than the minimum disparity difference the headset can deliver.

## Conclusions

We developed a VR environment where four balls served as a three-dimensional stimulus. One ball appears closer to the user, which has to be identified as fast as possible using the appropriate arrow key on a keyboard. Monocular depth cues were reduced, so the participant needed stereopsis to fulfill the task. Thus, the system could measure the stereopsis performance regarding reaction time and accuracy. Moreover, an eye tracking system recorded the pupil diameter and the gaze direction throughout the procedure. The raw data were preprocessed to cope with invalid or missing data. Therefrom, we extracted statistical features and features describing the individual changes in pupil diameter and gaze. We performed a feature selection method to evaluate each feature’s importance. However, it was not used to optimize for the best number of features. We collected data from 35 participants (15 controls and 20 Post-COVID) and trained different classifiers (SVM, kNN, and RF) on the features we extracted. Finally, we applied NCV with the inner loop for hyperparameter tuning the classifier model and the outer loop for the overall model performance. We achieved the best performance (71% accuracy) using the RF classifier. We provided features from eye tracking and stereopsis performance data in which differences can be observed, although not every Post-COVID patient showed these differences. Since the disease pattern is diffuse, not every Post-COVID patient is affected similarly. Furthermore, the sample size is small, only containing 35 participants. Therefore, we identified features showing differences between the groups. However, the rather small differences and the lack of sufficiently large datasets should be the subject of further investigations. We proposed a classification pipeline to distinguish controls from Post-COVID patients where we achieved an accuracy that might not be sufficient yet to be released. However, future work can improve on the discussed limitations of the system, and further data collection could support the presented findings. Nevertheless, these results indicate great potential for even better classification results.

Although the results seem promising, future work should assess this system in a large study including different age groups. Additionally, the participants were asked to take off their spectacles. This study limitation should be addressed in future work since stereoacuity relies on proper vision. By disabling corrections done by the spectacles, we might have introduced a reduction in their stereopsis performance, potentially distorting the resulting data. Furthermore, different feature types could be used in the classifiers, for example, features that consider the signals’ changes over time. Overall, we identified valuable features that seem prominent in Post-COVID patients and shed more light on how Post-COVID influences visual functions. We also proposed a classification pipeline for Post-COVID patients based on features from stereopsis and eye tracking data. Since neurophysiological dysfunctions occur not only in Post-COVID, this method might apply to other disorders like mild traumatic brain injuries that often occur in sports.

### Supplementary Information

Supplementary Information.

## Data Availability

The datasets generated during and/or analyzed during the current study are not publicly available due to data privacy restrictions but are available from the corresponding author on reasonable request.

## References

[1] Caldaria A (2020). COVID-19 and SARS: Differences and similarities. Dermatol. Ther..

[2] Hu B, Guo H, Zhou P, Shi Z-L (2021). Characteristics of SARS-CoV-2 and COVID-19. Nat. Rev. Microbiol..

[3] Dong E, Du H, Gardner L (2020). An interactive web-based dashboard to track COVID-19 in real time. Lancet Infect. Dis..

[4] Weekly epidemiological update on COVID-19-13 April 2023. https://www.who.int/publications/m/item/weekly-epidemiological-update-on-covid-19-13-april-2023 (Accessed 17 April 2023).

[5] *COVID-19 Rapid Guideline: Managing the Long-term Effects of COVID-19* (National Institute for Health and Care Excellence (NICE), 2020). 33555768

[6] Ahmad I, Rathore FA (2020). Neurological manifestations and complications of COVID-19: A literature review. J. Clin. Neurosci..

[7] Nalbandian A (2021). Post-acute COVID-19 syndrome. Nat. Med..

[8] Soriano JB, Murthy S, Marshall JC, Relan P, Diaz JV (2022). A clinical case definition of post-COVID-19 condition by a Delphi consensus. Lancet Infect. Dis..

[9] Koczulla AR (2022). S1-leitlinie long-/post-COVID. Pneumologie.

[10] Bliddal S (2021). Acute and persistent symptoms in non-hospitalized PCR-confirmed COVID-19 patients. Sci. Rep..

[11] Henneghan AM, Lewis KA, Gill E, Kesler SR (2022). Cognitive impairment in non-critical, mild-to-moderate COVID-19 survivors. Front. Psychol..

[12] Knight DRT (2022). Perception, prevalence, and prediction of severe infection and post-acute sequelae of COVID-19. Am. J. Med. Sci..

[13] Maltezou HC, Pavli A, Tsakris A (2021). Post-COVID syndrome: An insight on its pathogenesis. Vaccines.

[14] Carfì A, Bernabei R, Landi F (2020). Persistent symptoms in patients after acute COVID-19. JAMA.

[15] Fernández-de Las-Peñas C (2021). Prevalence of post-COVID-19 symptoms in hospitalized and non-hospitalized COVID-19 survivors: A systematic review and meta-analysis. Eur. J. Intern. Med..

[16] Lonkar BK (2021). A review on post COVID-19 effects. Int. J. Pharm. Pharmacol..

[17] Scordo KA, Richmond MM, Munro N (2021). Post-COVID-19 syndrome: Theoretical basis, identification, and management. AACN Adv. Crit. Care.

[18] Silva Andrade B (2021). Long-COVID and post-COVID health complications: An up-to-date review on clinical conditions and their possible molecular mechanisms. Viruses.

[19] Zhao S (2022). Rapid vigilance and episodic memory decrements in COVID-19 survivors. Brain Commun..

[20] Lu Y (2020). Cerebral micro-structural changes in COVID-19 patients—an MRI-based 3-month follow-up study. EClinicalMedicine.

[21] Voruz P (2022). Long COVID neuropsychological deficits after severe, moderate, or mild infection. Clin. Transl. Neurosci..

[22] Wu Y (2020). Nervous system involvement after infection with COVID-19 and other coronaviruses. Brain Behav. Immunity.

[23] Huang C (2021). 6-month consequences of COVID-19 in patients discharged from hospital: A cohort study. Lancet.

[24] Shanley JE (2022). Longitudinal evaluation of neurologic-post acute sequelae SARS-CoV-2 infection symptoms. Ann. Clin. Transl. Neurol..

[25] Crivelli L (2022). Changes in cognitive functioning after COVID-19: A systematic review and meta-analysis. Alzheimer’s Dement..

[26] García Cena C (2022). Eye movement alterations in post-COVID-19 condition: A proof-of-concept study. Sensors.

[27] Wennmo C, Hindfelt B (1980). Eye movements in brainstem lesions. Acta oto-laryngologica.

[28] Bitirgen G (2022). Abnormal quantitative pupillary light responses following COVID-19. Int. Ophthalmol..

[29] Yurttaser Ocak S, Ozturan SG, Bas E (2022). Pupil responses in patients with COVID-19. Int. Ophthalmol..

[30] Chen C (2022). Global prevalence of post COVID-19 condition or long COVID: A meta-analysis and systematic review. J. Infect. Dis..

[31] Oronsky B (2021). A review of persistent post-COVID syndrome (PPCS). Clin. Rev. Allergy Immunol..

[32] Fugazzaro S (2022). Rehabilitation interventions for post-acute COVID-19 syndrome: A systematic review. Int. J. Environ. Res. Public Health.

[33] de Sire A (2022). Impact of rehabilitation on fatigue in post-COVID-19 patients: A systematic review and meta-analysis. Appl. Sci..

[34] Rovere Querin P (2020). Post-COVID-19 follow-up clinic: Depicting chronicity of a new disease. Acta bio-medica: Atenei Parmensis.

[35] Solomon JJ, Heyman B, Ko JP, Condos R, Lynch DA (2021). CT of post-acute lung complications of COVID-19. Radiology.

[36] Yelin D (2022). ESCMID rapid guidelines for assessment and management of long COVID. Clin. Microbiol. Infect..

[37] Samuel AL (1959). Some studies in machine learning using the game of checkers. IBM J. Res. Dev..

[38] Kara DD, Ring M, Hennig FF, Michelson G (2020). Effects of mild traumatic brain injury on stereopsis detected by a virtual reality system: Attempt to develop a screening test. J. Med. Biol. Eng..

[39] Mehringer W, Wirth M, Roth D, Michelson G, Eskofier BM (2022). Stereopsis only: Validation of a monocular depth cues reduced gamified virtual reality with reaction time measurement. IEEE Trans. Vis. Comput. Graph..

[40] Paulus J, Hornegger J, Schmidt M, Eskofier B, Michelson G (2012). Novel stereo vision test for far distances measuring perception time as a function of disparity in a virtual environment. Investig. Ophthalmol. Vis. Sci..

[41] Paulus, J. *et al.* Measurement of individual changes in the performance of human stereoscopic vision for disparities at the limits of the zone of comfortable viewing. In *Proceedings-2013 International Conference on 3D Vision, 3DV 2013*, 310–317, 10.1109/3DV.2013.48 (2013).

[42] Paulus J (2014). Extended stereopsis evaluation of professional and amateur soccer players and subjects without soccer background. Front. Psychol..

[43] Schoemann MD, Lochmann M, Paulus J, Michelson G (2017). Repetitive dynamic stereo test improved processing time in young athletes. Restor. Neurol. Neurosci..

[44] Abunadi I (2022). An automated glowworm swarm optimization with an inception-based deep convolutional neural network for COVID-19 diagnosis and classification. Healthcare.

[45] Phetsouphanh C (2022). Immunological dysfunction persists for 8 months following initial mild-to-moderate SARS-CoV-2 infection. Nat. Immunol..

[46] Khan M (2021). Applications of artificial intelligence in COVID-19 pandemic: A comprehensive review. Expert Syst. Appl..

[47] Meraihi Y, Gabis AB, Mirjalili S, Ramdane-Cherif A, Alsaadi FE (2022). Machine learning-based research for COVID-19 detection, diagnosis, and prediction: A survey. SN Comput. Sci..

[48] Kermali M, Khalsa RK, Pillai K, Ismail Z, Harky A (2020). The role of biomarkers in diagnosis of COVID-19—A systematic review. Life Sci..

[49] Gaber T (2021). Assessment and management of post-COVID fatigue. Prog. Neurol. Psychiatry.

[50] Aidar MN, Gomes TM, de Almeida MZH, de Andrade EP, Serracarbassa PD (2021). Low visual acuity due to acute macular neuroretinopathy associated with COVID-19: A case report. Am. J. Case Rep..

[51] Conrady CD, Faia LJ, Gregg KS, Rao RC (2021). Coronavirus-19-associated retinopathy. Ocul. Immunol. Inflamm..

[52] Jevnikar K, Jaki Mekjavic P, Vidovic Valentincic N, Petrovski G, Globocnik Petrovic M (2021). An update on COVID-19 related ophthalmic manifestations. Ocul. Immunol. Inflamm..

[53] Lecler A, Cotton F, Lersy F, Kremer S, Héran F (2021). Ocular MRI findings in patients with severe COVID-19: A retrospective multicenter observational study. Radiology.

[54] Montesel A, Bucolo C, Mouvet V, Moret E, Eandi CM (2020). Case report: Central retinal artery occlusion in a COVID-19 patient. Front. Pharmacol..

[55] Sen S (2022). Retinal manifestations in patients with SARS-CoV-2 infection and pathogenetic implications: A systematic review. Int. Ophthalmol..

[56] Soltani S (2022). Pooled prevalence estimate of ocular manifestations in COVID-19 patients: A systematic review and meta-analysis. Iran. J. Med. Sci..

[57] Szczesniak M, Brydak-Godowska J (2021). SARS-CoV-2 and the eyes: A review of the literature on transmission, detection, and ocular manifestations. Med. Sci. Monit. Int. Med. J. Exp. Clin. Res..

[58] Brantl V (2021). Long-term ocular damage after recovery from COVID-19: Lack of evidence at three months. BMC Ophthalmol..

[59] Yong SJ (2021). Persistent brainstem dysfunction in long-COVID: A hypothesis. ACS Chem. Neurosci..

[60] Jennings G, Monaghan A, Xue F, Duggan E, Romero-Ortuño R (2022). Comprehensive clinical characterisation of brain fog in adults reporting long COVID symptoms. J. Clin. Med..

[61] Vyas A (2022). Mild cognitive impairment in COVID-19 survivors: Measuring the brain fog. Int. J. Ment. Health.

[62] Lynch S (2022). Screening for brain fog: Is the Montreal cognitive assessment an effective screening tool for neurocognitive complaints post-COVID-19?. Gen. Hosp. Psychiatry.

[63] Howard IP, Rogers BJ (2012). Perceiving in Depth Volume 2 Stereoscopic Vision.

[64] Neumann DL (2018). A systematic review of the application of interactive virtual reality to sport. Virtual Real..

[65] Michelson, G. Verfahren zur erfassung der zerebralen kognitionszeit sowie vorrichtung zur erfassung der zerebralen kognitionszeit (2018).

[66] Michelson, G. Method and device for quantitatively detecting the fusion capacity in conjugate eye movements (2021).

[67] Paulus, J. *Evaluation Methods for Stereopsis Performance*. Doctoralthesis, Friedrich-Alexander-Universität Erlangen-Nürnberg (FAU) (2016).

[68] Bell DS (1994). The Doctor’s Guide to Chronic Fatigue Syndrome: Understanding, Treating, and Living with CFIDS.

[69] Stoeve M (2022). Eye tracking-based stress classification of athletes in virtual reality. Proc. ACM Comput. Graph. Interact. Tech..

[70] Kret ME, Sjak-Shie EE (2019). Preprocessing pupil size data: Guidelines and code. Behav. Res. Methods.

[71] Alsaeedi N, Wloka D (2021). Velocity-dependent perception threshold for discrete imperceptible repositioning in a virtual environment during eye blinks. IEEE Access.

[72] Dan, E. L., Dinsoreanu, M. & Muresan, R. C. Accuracy of six interpolation methods applied on pupil diameter data. In *2020 IEEE International Conference on Automation, Quality and Testing, Robotics (AQTR)*, 1–5, 10.1109/AQTR49680.2020.9129915 (IEEE, 2020).

[73] Bishop CM (2006). Pattern Recognition and Machine Learning. Information Science and Statistics.

[74] Imaoka Y, Flury A, de Bruin ED (2020). Assessing saccadic eye movements with head-mounted display virtual reality technology. Front. Psychiatry.

[75] Tirdad K (2021). Machine learning-based approach to analyze saccadic eye movement in patients with mild traumatic brain injury. Comput. Methods Progams Biomed. Update.

[76] Vodrahalli, K., Filipkowski, M., Chen, T., Zou, J. & Liao, Y. J. Predicting visuo-motor diseases from eye tracking data. In *Pacific Symposium on Biocomputing. Pacific Symposium on Biocomputing* Vol. 27, 242–253 (2022). PMC10635679 34890153

[77] Yaneva V, Le H, Eraslan S, Yesilada Y, Mitkov R (2020). Detecting high-functioning autism in adults using eye tracking and machine learning. IEEE Trans. Neural Syst. Rehabil. Eng..

[78] Duchowski, A. T., Krejtz, K., Gehrer, N. A., Bafna, T. & Bækgaard, P. The low/high index of pupillary activity. In In *Proceedings of the 2020 CHI Conference on Human Factors in Computing Systems*, (eds Bernhaupt, R. *et al.*) 1–12, 10.1145/3313831.3376394 (ACM, 2020).

[79] Baltaci S, Gokcay D (2016). Stress detection in human–computer interaction: Fusion of pupil dilation and facial temperature features. Int. J. Hum.-Comput. Interact..

[80] DiCriscio AS, Hu Y, Troiani V (2019). Brief report: Visual perception, task-induced pupil response trajectories and ASD features in children. J. Autism Dev. Disord..

[81] Mathôt S (2018). Pupillometry: Psychology, physiology, and function. J. Cogn..

[82] Sabatino DiCriscio A, Hu Y, Troiani V (2018). Task-induced pupil response and visual perception in adults. PLoS ONE.

[83] Winn MB, Wendt D, Koelewijn T, Kuchinsky SE (2018). Best practices and advice for using pupillometry to measure listening effort: An introduction for those who want to get started. Trends Hear..

[84] Duchowski, A. T. *et al.* 3d eye movement analysis for vr visual inspection training. In *Proceedings of the symposium on Eye tracking research & applications-ETRA ’02*, (eds Duchowski, A. T. *et al.*) 103, 10.1145/507072.507094 (ACM Press, 2002).

[85] Cortes C, Vapnik V (1995). Support-vector networks. Mach. Learn..

[86] Breiman L (2001). Random forests. Mach. Learn..

[87] Dasarathy, B. V. Nearest neighbor (NN) norms: NN pattern classification techniques. In (ed. Dasarathy, B. V.) 1st edn. (IEEE Computer Society Press, 1991).

[88] Pedregosa F (2011). Scikit-learn: Machine learning in Python. J. Mach. Learn. Res..

[89] Saeb S, Lonini L, Jayaraman A, Mohr DC, Kording KP (2017). The need to approximate the use-case in clinical machine learning. GigaScience.

[90] Aiyegbusi OL (2021). Symptoms, complications and management of long COVID: A review. J. R. Soc. Med..

[91] Montes-Ibarra M (2022). The impact of long COVID-19 on muscle health. Clin. Geriatr. Med..

[92] Tolosi L, Lengauer T (2011). Classification with correlated features: Unreliability of feature ranking and solutions. Bioinformatics.

[93] Rani R, Khurana M, Kumar A, Kumar N (2022). Big data dimensionality reduction techniques in IoT: Review, applications and open research challenges. Cluster Comput..

[94] Huang X, Wu L, Ye Y (2019). A review on dimensionality reduction techniques. Int. J. Pattern Recogn. Artif. Intell..

[95] Hamilton JD (1994). Time Series Analysis.

[96] Nielsen A (2021). Practical Time Series Analysis: Prediction with Statistics and Machine Learning.

[97] Clay V, König P, König S (2019). Eye tracking in virtual reality. J. Eye Mov. Res..

[98] Carter BT, Luke SG (2020). Best practices in eye tracking research. Int. J. Psychophysiol..

[99] Hultsch DF, MacDonald SWS, Dixon RA (2002). Variability in reaction time performance of younger and older adults. J. Gerontol. Ser. B Psychol. Sci. Soc. Sci..

[100] Wright LA, Wormald RP (1992). Stereopsis and ageing. Eye.

[101] Olson KE, O’Brien MA, Rogers WA, Charness N (2011). Diffusion of technology: Frequency of use for younger and older adults. Ageing Int..

[102] Perlis, R. H. *et al.* Persistence of symptoms up to 10 months following acute COVID-19 illness. *medRxiv : the preprint server for health sciences*10.1101/2021.03.07.21253072 (2021).

[103] O’Mahoney LL (2023). The prevalence and long-term health effects of long COVID among hospitalised and non-hospitalised populations: A systematic review and meta-analysis. EClinicalMedicine.

[104] von Noorden, G. K. & Campos, E. C. *Binocular Vision and Ocular Motility: Theory and Management of Strabismus/Gunter K. von Noorden, Emilio C. Campos* 6th ed (Mosby, 2001).

