# Author Correction: Virtual reality for assessing stereopsis performance and eye characteristics in Post-COVID

**DOI:** 10.1038/s41598-023-43373-7

**Published:** 2023-09-28

**Authors:** Wolfgang Mehringer, Maike Stoeve, Daniel Krauss, Matthias Ring, Fritz Steussloff, Moritz Güttes, Julia Zott, Bettina Hohberger, Georg Michelson, Bjoern Eskofier

**Affiliations:** 1 Machine Learning and Data Analytics Lab (MaD Lab), Department Artificial Intelligence in Biomedical Engineering (AIBE), Friedrich-Alexander-Universität Erlangen-Nürnberg (FAU), 91052 Erlangen, Bavaria Germany; 2 Department of Ophthalmology, Universitätsklinikum Erlangen, Friedrich-Alexander-Universität Erlangen-Nürnberg, 91054 Erlangen, Germany; 3 Talkingeyes & More GmbH, 91052 Erlangen, Bavaria Germany

Correction to: *Scientific Reports* 10.1038/s41598-023-40263-w, published online 13 August 2023

In the original version of this Article, Bettina Hohberger, Georg Michelson and Bjoern Eskofier were omitted as equally contributing authors.

The original Article has been corrected.

