# Supplementary material for: Virtual reality for assessing stereopsis performance and eye characteristics in Post-COVID
Source: Sci Rep. 2023 Aug 13;13:13167. doi: 10.1038/s41598-023-40263-w (PMC10423723; doi:10.1038/s41598-023-40263-w)
Supplement: Supplementary file 1 — Supplementary Information. [file 41598_2023_40263_MOESM1_ESM.pdf]

Supplementary Material for Virtual Reality for Assessing Stereopsis Performance and Eye Characteristics in Post-COVID

Wolfgang Mehringer[1,*], Maike Stoeve[1], Daniel Krauss[1], Matthias Ring[1], Fritz Steussloff[2], Moritz Güttes[2], Julia Zott[2], Bettina Hohberger[2], Georg Michelson[2,3], and Bjoern Eskofier[1]

[1]Machine Learning and Data Analytics Lab (MaD Lab), Department Artificial Intelligence in Biomedical Engineering (AIBE), Friedrich-Alexander-Universität Erlangen-Nürnberg (FAU), Erlangen, 91052, Bavaria, Germany,
[2]Department of Ophthalmology, Friedrich-Alexander-Universität Erlangen-Nürnberg (FAU), Erlangen, 91054, Bavaria, Germany
[3]Talkingeyes & More GmbH, Erlangen, 91052, Bavaria, Germany
[*]wolfgang.mehringer@fau.de

ABSTRACT

In 2019, we faced a pandemic due to the coronavirus disease (COVID-19), with millions of confirmed cases and reported deaths. Even in recovered patients, symptoms can be persistent over weeks, termed Post-COVID. In addition to common symptoms of fatigue, muscle weakness, and cognitive impairments, visual impairments have been reported. Automatic classification of COVID and Post-COVID is researched based on blood samples and radiation-based procedures, among others. However, a symptom-oriented assessment for visual impairments is still missing. Thus, we propose a Virtual Reality environment in which stereoscopic stimuli are displayed to test the patient's stereopsis performance. While performing the visual tasks, the eyes' gaze and pupil diameter are recorded. We collected data from 15 controls and 20 Post-COVID patients in a study. Therefrom, we extracted features of three main data groups, stereopsis performance, pupil diameter, and gaze behavior, and trained various classifiers. The Random Forest (RF) classifier achieved the best result with 71% accuracy. The data support the classification result showing worse stereopsis performance and eye movement alterations in Post-COVID. This system might help the overburdened healthcare system by supporting the diagnosis of exclusion and automatically monitoring the progress of Post-COVID.

Data Groups & Features

This section of the supplementary materials provides visualizations (Fig. 1, Fig. 2, and Fig. 3) of all available features split into the different data groups. The features were all normalized for better comparison and to represent the input to the classifiers. For normalization, we extracted the minimum and maximum in each feature dataset and applied the following scaling.

$$X_{norm} = \frac{X - X_{min}}{X_{min} - X_{max}} \tag{1}$$

X represents a data point in the feature dataset. X_{min} represents the minimum or smallest data point, and X_{max} the maximum or largest data point in the feature dataset.

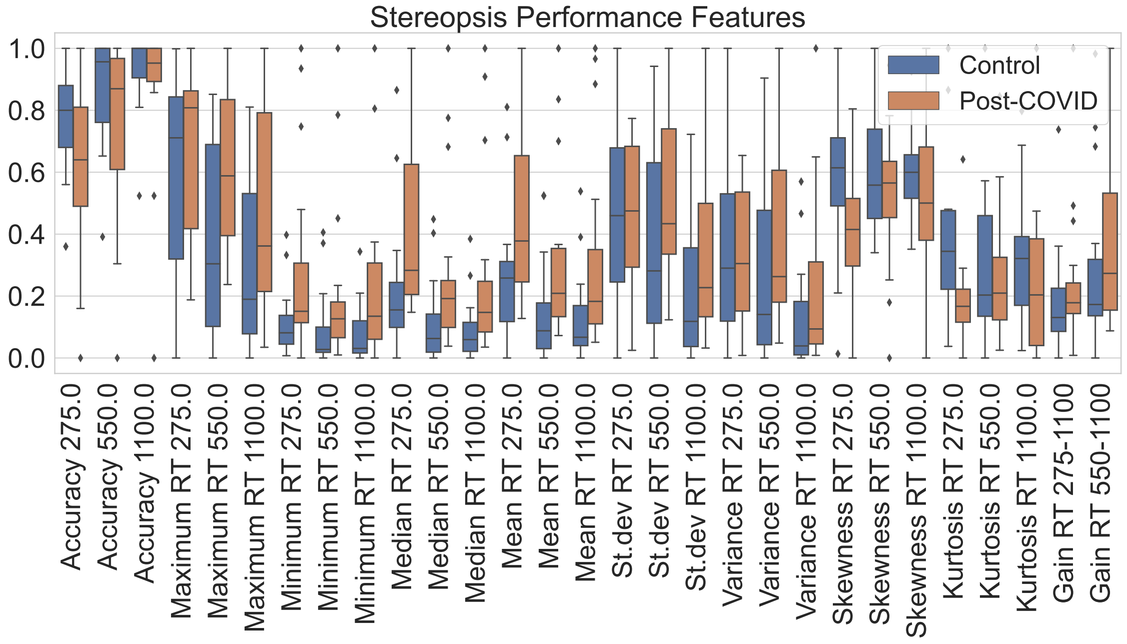

Figure 1. The 29 features for the *stereopsis performance* data are shown. The features were normalized for better comparison and to bring them into the same value range. The abbreviation *RT* refers to Reaction Time, hinting at the source of the feature. The number denotes the disparity setting. The features presented here were also used as input for the classifiers.

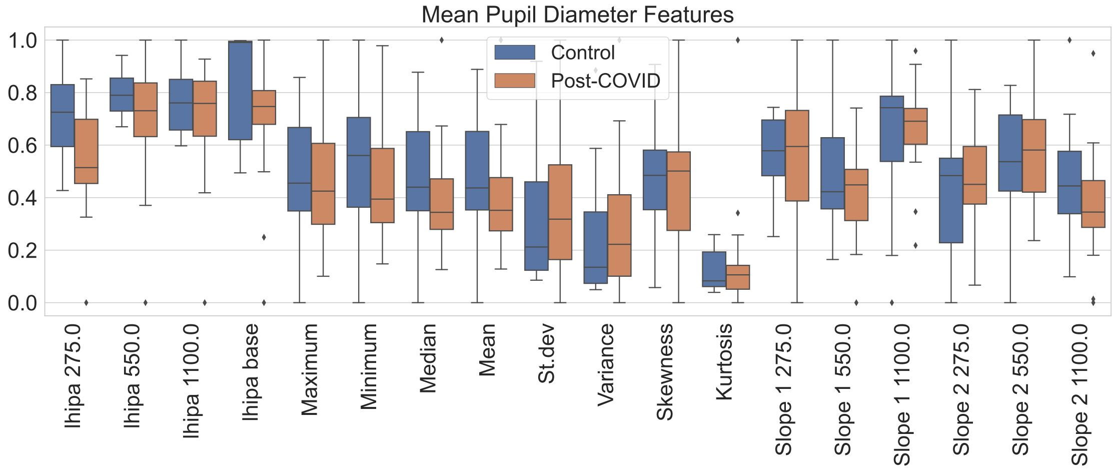

Figure 2. The twelve features for the *pupil diameter* data are shown. Each feature is based on the mean pupil diameter signal for the left and right eye. The LHIPA value was divided into the disparity setting (given by the number) and a base value calculated one second before a disparity was set. The features were also normalized and used as input for the classifiers.

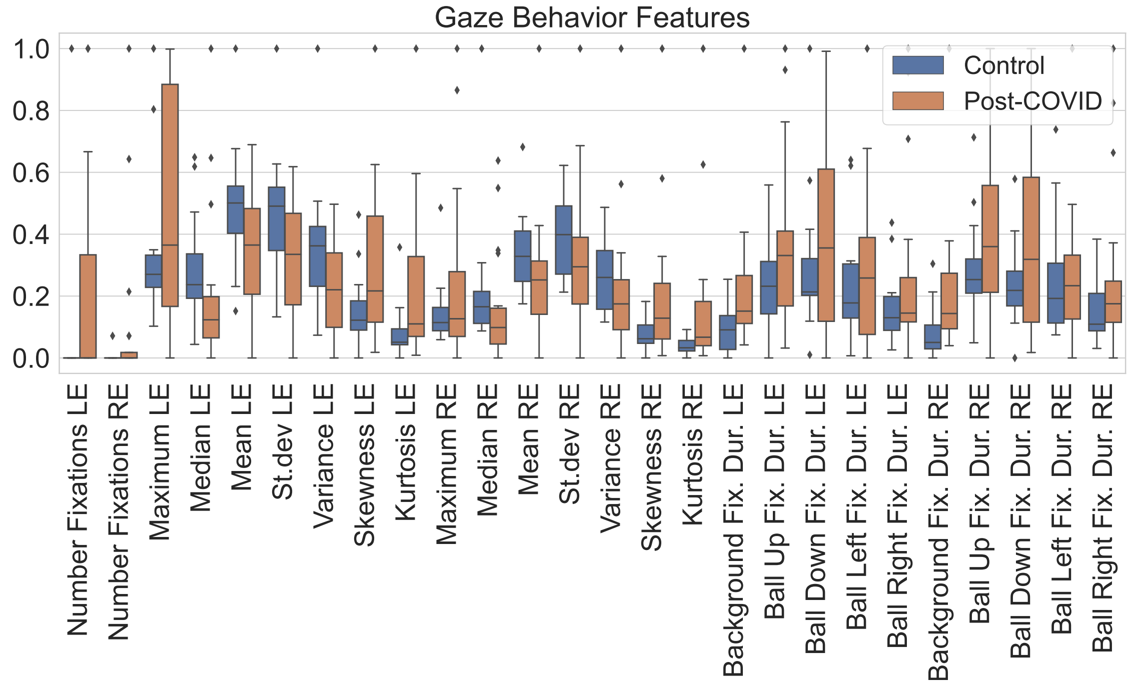

Figure 3. The 26 features for the *gaze behavior* data are shown. The abbreviations *LE* and *RE* describe the left and right eye, respectively. The abbreviation *Fix. Dur.* means Fixation Duration. Again, the features were normalized and taken as input for the classifiers.
